# Immunotherapy-Induced Hepatitis Mimicking Sclerosing Cholangitis in a Patient With Metastatic Triple-Negative Breast Cancer

**DOI:** 10.7759/cureus.92515

**Published:** 2025-09-17

**Authors:** Mohammed Khizer, Mohammad Faize, Mohammad Hamza, Saeed Omar

**Affiliations:** 1 Internal Medicine, Glenfield Hospital, Leicester, GBR; 2 Gastroenterology, Leicester Royal Infirmary, Leicester, GBR; 3 Gastroenterology and Hepatology, Leicester Royal Infirmary, Leicester, GBR

**Keywords:** atezolizumab, immune-checkpoint inhibitors, liver toxicity, sclerosing cholangitis, triple-negative breast cancer (tnbc)

## Abstract

Immune checkpoint inhibitors (ICIs) have emerged as a promising treatment for metastatic triple-negative breast cancer (TNBC), particularly in programmed death-ligand 1 (PD-L1)-positive tumors. However, their use is associated with immune-related adverse events (irAEs), including hepatotoxicity, which, although rare, can be severe and challenging to diagnose. We present the case of a 59-year-old woman with metastatic TNBC who developed immune-related liver toxicity following 14 cycles of atezolizumab in combination with nab-paclitaxel. The patient presented with jaundice, pruritus, and transaminitis. Liver biopsy revealed nonspecific findings, including interface hepatitis and early fibrosis, without features definitive for sclerosing cholangitis. A comprehensive workup excluded viral, autoimmune, and other drug-induced causes. Based on clinical timing, exclusion of alternative etiologies, and histopathologic correlation, a diagnosis of ICI-induced hepatitis was made. The patient responded well to corticosteroid therapy with budesonide, showing gradual improvement in liver enzyme levels. This case underscores the importance of maintaining a high index of suspicion for immune-related hepatotoxicity in patients receiving ICIs, especially those with underlying liver conditions, and highlights the need for timely diagnosis and individualized immunosuppressive treatment.

## Introduction

Triple-negative breast cancer (TNBC) is an aggressive subtype of breast cancer characterized by the absence of estrogen receptor (ER), progesterone receptor (PR), and human epidermal growth factor receptor 2 (HER2) expression. TNBC accounts for approximately 10%-20% of all breast cancer cases and is associated with higher rates of recurrence, metastasis, and mortality compared to other subtypes [1,2]. While chemotherapy has historically been the backbone of TNBC treatment, the advent of immune checkpoint inhibitors (ICIs) has opened new therapeutic avenues, particularly for patients with programmed death-ligand 1 (PD-L1)-positive disease [3,4].

Atezolizumab, a programmed death-ligand 1 (PD-L1) inhibitor, in combination with nab-paclitaxel, was the first immunotherapy approved for metastatic TNBC, demonstrating significant improvements in progression-free survival for PD-L1-positive tumors [4,5]. Additionally, other studies, such as the KEYNOTE-355 trial, have demonstrated that Pembrolizumab plus chemotherapy is an effective treatment for previously untreated metastatic TNBC [6]. PD-L1 expression plays a critical role in suppressing T-cell-mediated anti-tumor immunity by binding to the programmed cell death protein 1 (PD-1) receptor on T cells. ICIs such as atezolizumab and pembrolizumab block this interaction, restoring T-cell activity and enhancing anti-tumor immune responses. While this mechanism underlies their therapeutic benefit, it also explains the occurrence of immune-related adverse events due to systemic immune activation. However, the use of ICIs is associated with immune-related adverse events (irAEs), which result from nonspecific activation of the immune system. Among these, hepatotoxicity, although less common than dermatologic or gastrointestinal irAEs, is a potentially severe complication [7].

Immune-related hepatitis occurs in approximately 1%-6% of patients treated with ICIs and can present as asymptomatic transaminitis or severe immune-mediated liver injury [8]. Risk factors for ICI-induced hepatitis include pre-existing liver disease, combination immunotherapy, and high tumor burden. Histological findings typically include lobular inflammation, hepatocyte necrosis, and infiltration by activated T cells and other immune cells [9,10]. Diagnostic challenges arise due to overlapping features with other causes of liver dysfunction, such as viral hepatitis, drug-induced liver injury, or autoimmune liver diseases.

Management of immune-related hepatitis involves prompt discontinuation of ICIs and initiation of immunosuppressive therapy, such as corticosteroids. In cases refractory to steroids, additional immunosuppressants, including mycophenolate mofetil or tacrolimus, may be required. While most patients recover with timely intervention, delays in recognition or treatment can result in progression to liver failure. In certain cases, budesonide may be preferred over prednisone because of its high first-pass hepatic metabolism, which allows targeted action within the liver while minimizing systemic side effects.

This case report presents a patient with metastatic TNBC who developed immune-related hepatitis following 14 cycles of atezolizumab and nab-paclitaxel. The case highlights the challenges of diagnosing and managing ICI-induced hepatotoxicity, particularly in the context of pre-existing liver conditions. Our aim is to underscore the need for vigilant monitoring and individualized management of irAEs in patients undergoing ICI therapy.

## Case presentation

A 59-year-old female with metastatic TNBC presented with jaundice, pruritus, and dark urine after receiving 14 cycles over seven months of atezolizumab and nab-paclitaxel. Her medical history included drug-induced chronic hepatitis and carcinoma in situ of the breast. Liver function tests showed progressive elevation in alanine aminotransferase (ALT), peaking at 609 IU/L. Ultrasound of the liver was performed, which showed benign hepatic cysts without malignant features.

**Figure 1:**
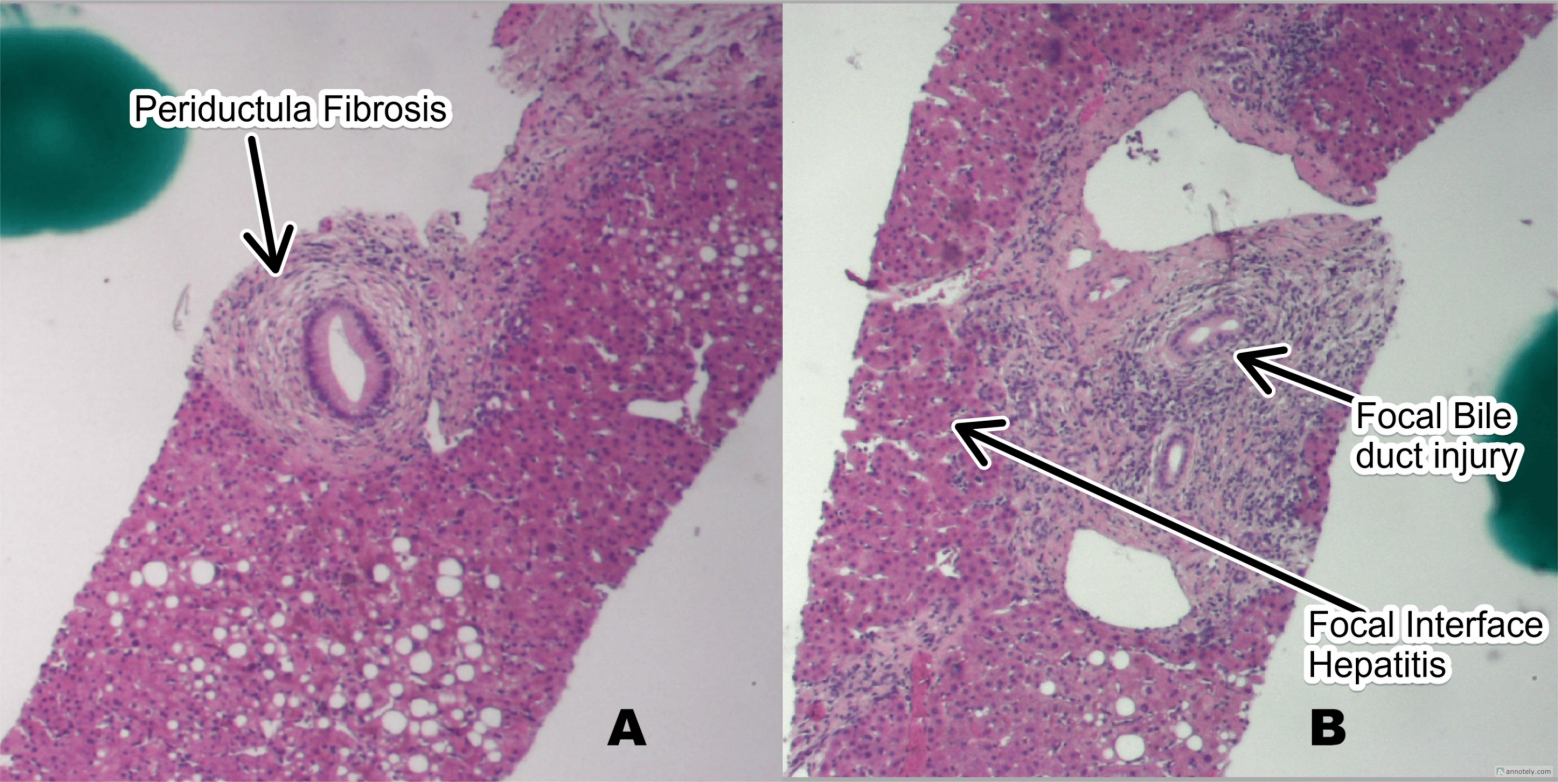
Liver Biopsy Demonstrating Focal Bile Duct Injury and Early Fibrosis With Interface Hepatitis (H&E Stain) These images 'A' and 'B' show focal bile duct injury in the liver biopsy of a 59-year-old female with suspected immune checkpoint inhibitor-induced hepatitis. The injury is characterized by localized bile duct damage, with an inflammatory infiltrate around the bile ducts. The histopathological features are not consistent with sclerosing cholangitis secondary to immunotherapy, as per re-review by the pathologist, and suggest a nonspecific injury pattern. H&E: hematoxylin and eosin stain.

A liver biopsy was performed to evaluate the etiology of liver dysfunction. Histological analysis revealed focal bile duct injury, focal interface hepatitis, and early fibrosis, along with the presence of ceroid pigment in Kupffer cells and grade 1 iron deposition in hepatocytes. Copper-associated protein was noted, but no evidence of alpha-1-antitrypsin deficiency or viral hepatitis was found. Based on a re-review by the pathologist, the histologic pattern was not consistent with sclerosing cholangitis secondary to immunotherapy. The changes were nonspecific and can be seen in a range of liver pathologies, necessitating a clinical correlation (Figures 1A, 1B).

Comprehensive viral and autoimmune liver screens were negative or non-contributory, except for positive Epstein-Barr virus (EBV) serology, indicating past or reactivated infections. Autoimmune markers including antinuclear antibody (ANA), smooth muscle antibody (SMA), and liver kidney microsome type 1 antibody (LKM-1) were negative. The simplified autoimmune hepatitis score was considered but not calculated given histology inconsistent with autoimmune hepatitis. Given the timing of symptom onset, exclusion of alternative causes, and histological pattern, a clinical diagnosis of ICI-induced hepatitis was made. Atezolizumab and nab-paclitaxel were discontinued (Table 1).

**Table 1:** Viral and Hepatitis Serology Results During Workup for Liver Dysfunction This table summarizes the results of viral and hepatitis screening conducted to evaluate the etiology of liver enzyme elevation. Serology showed evidence of past or reactivated Epstein-Barr virus (EBV) infection and prior exposure to hepatitis A virus (positive total HAV antibodies with negative IgM), suggesting no acute viral hepatitis. Tests for acute hepatitis B, C, and E, as well as cytomegalovirus (CMV), were negative. EBNA: Epstein-Barr nuclear antigen, VCA: viral capsid antigen, HAV: hepatitis A virus.

Test	Result
Antibodiesto Hepatitis A (Total)	Detected
Hepatitis A IgM	Negative
Hepatitis B Surface Antigen (HBsAg)	Negative
Hepatitis B Core Antibody	Negative
Hepatitis E IgM	Negative
Hepatitis C Antibody	Negative
Cytomegalovirus (CMV) IgM	Negative
Epstein-Barr Virus (EBV) EBNA IgG	Positive
EBV VCA IgM	Positive
EBV VCA IgG	Positive

The patient was managed with supportive therapy and corticosteroids. Budesonide was commenced in November 2024 at a dose of 9 mg daily. ALT levels peaked just prior to initiation and then showed a clear downward trend in the following days and weeks. Budesonide was chosen over prednisone for its targeted hepatic effect and favorable safety profile in the context of pre-existing chronic liver disease. Therapy was continued for 90 days and tapered to 3 mg daily as maintenance (Table 2).

**Table 2:** ALT Levels Table showing the change in serum ALT levels. ALT levels peaked at 609 IU/L during acute liver dysfunction and gradually declined to 26 IU/L over the course of corticosteroid (budesonide) therapy. The trend reflects a sustained biochemical response to immunosuppressive treatment. ALT: alanine aminotransferase. Reference range: 2-53. IU/L: international units per liter.

Dates	ALT (IU/L)
26-09-24	124
03-10-24	274
10-10-24	431
23-10-24	569
24-10-24	559
26-10-24	589
03-11-24	305
09-11-24	609
10-11-24	596
11-11-24	458
12-11-24	406
13-11-24	307
15-11-24	231
23-11-24	65
07-12-24	26

## Discussion

Immune checkpoint inhibitors (ICIs), such as atezolizumab, have significantly improved outcomes in metastatic triple-negative breast cancer (TNBC), particularly in PD-L1-positive patients. However, their use is associated with immune-related adverse events (irAEs), among which hepatotoxicity is a rare but potentially severe complication. In this case, a 59-year-old woman developed immune-related liver toxicity after 14 cycles of atezolizumab and nab-paclitaxel.

The patient presented with jaundice, pruritus, and elevated liver enzymes, prompting a liver biopsy, which revealed focal bile duct injury, interface hepatitis, and early fibrosis. These findings were nonspecific and could be seen in various liver pathologies, including drug-induced liver injury, autoimmune hepatitis, and viral hepatitis. The absence of features consistent with sclerosing cholangitis ruled out this as a definitive diagnosis. Given the timing of the patient’s symptoms and the exclusion of other potential causes through comprehensive workup, a diagnosis of ICI-induced hepatitis was established. The patient responded well to budesonide, a corticosteroid, with gradual improvement in liver function, reinforcing the clinical diagnosis.

This case underscores the diagnostic challenges of immune-related hepatotoxicity, where liver biopsy findings may be nonspecific. Despite the histological overlap with other liver diseases, the clinical context and timing of symptoms are critical in making an accurate diagnosis. Furthermore, the case highlights the importance of early recognition and intervention in preventing irreversible liver damage. The patient’s favorable response to steroid therapy reinforces the efficacy of individualized treatment for ICI-induced hepatotoxicity.

## Conclusions

This case highlights the complexities in diagnosing immune-related hepatotoxicity associated with ICIs, particularly when liver histology shows nonspecific changes. Although histology did not support a definitive diagnosis of sclerosing cholangitis, the clinical presentation, timing of symptoms, and exclusion of other etiologies strongly supported a diagnosis of immunotherapy-induced hepatitis, which behaved like sclerosing cholangitis. Budesonide was successfully used as targeted immunosuppressive therapy, with sustained normalization of liver enzymes. The case underscores the need for a multidisciplinary approach, combining histopathological findings with clinical judgment and vigilance for immune-related adverse events in patients with pre-existing liver disease undergoing ICI therapy.
